# An Ague Endemic at Cannington

**Published:** 1858-10

**Authors:** Alfred Haviland

**Affiliations:** Member of the Royal College of Surgeons, and of the British Meteorological and Epidemiological Societies.


### AGUE EPIDEMIC AT CANNINGTON.

By ALFRED HAVILAND, Member of the Royal College of Surgeons, and of the British Meteorological and Epidemiological Societies.

WHEN an epidemic suddenly reappears in a locality which it has not visited for many years, it behoves all, from the physician to the peasant, to meet the foe with a determined front, and join heart and hand in expelling it. In most instances, the extremities of this social chain would have no hesitation in combating the evil, but, unfortunately for humanity, there are links between, which are weakened by ignorance and prejudice, and so it often happens that the warning of the physician is unheeded and the prayer of the poor man is uncared for, whilst a chattering ratepayer is listened to as an oracle, in his attempt to ignore the calamity and its cause, and to prove that foul drains, and paupers debilitated and incapacitated for work by disease, are less expensive to the parish than clean drains and a healthy peasantry.

I mean these to be general remarks—*qui capit, ille facit.*

Cannington is a pretty little village in Somersetshire, situated about three miles north-west from Bridgewater, and about a mile and a quarter south-west from the river Parrett, which limits the alluvial plain, on whose western boundary the lower portion of the village is built; the majority of the houses to the west having for their foundation the red sandstone, and to the north-west the cottages lie upon the mountain limestone, which crops out in a most remarkable manner, forming the hill called Cannington Park. The village itself is sheltered from the north-westerly, the northerly, and the north-easterly winds, by the elevated red sandstone ridge, which rears itself to the north. Cannington gently slopes to its southern boundary,—a beautiful stream of water, derived from the Quantock Hills, and flowing into the Parrett, after forming an important drain for the marsh lands to the north-east. This stream supplies the mills at the higher portion of the village, and is capable of being turned off for the purpose of flushing the main drain. This, however, has never been done. Why?—The parishioners alone can answer. Through the centre of the village is a main drain, in a deplorable state of inefficiency, and a partial drain through the churchyard, which is on gravel, and during wet seasons admits much water, causing great inconvenience at times, after continued rains, to the gravedigger, and much *malaise* in those who drink the water of the neighbouring wells, into which that derived from the churchyard soaks, after leaving the putrescent bodies of its occupants. Nature has been kinder to Cannington than man; and if her gifts, viz., a beautiful air and a plentiful supply of water, were accepted and appreciated as they ought to be by the recipients of her bounty, this picturesque little village would rank still higher than it already does in the scale of healthiness : as it is, however, a total neglect of all hygienic rules renders its inhabitants liable to several epidemic fevers, which perchance, at no short date, may give them a severer lesson than they yet have had ; and then, and I fear not until then, will they look into those matters which so deeply concern themselves and their dependents. The remarkable feature of the present epidemic is its reappearance after an almost entire absence for a great number of years. About thirty or thirty-five years ago, the land around Cannington was badly drained and the ague was quite endemic. Within the last ten or twelve years, however, since the draining of land has been much in vogue, I have found only about one and a half per cent. of the cases admitted at the Cannington Dispensary to be those of ague. I was therefore surprised to find this small number leap up to nineteen per cent., and among the cases of illness occurring in two of the friendly societies, to twenty-nine per cent. I immediately set to work to investigate the cause, and found that not *undrained land,* as heretofore, was the *fons et origo mali,* but *undrained houses,* which the remarkable dryness of the season had assisted in rendering pestiferous. The Committee of Health, which had been formed at my suggestion in the November of 1857, when cholera was anticipated, had confined their labours to a solitary perambulation of the parish, which resulted in not a single nuisance being removed, or even an official order for that purpose given. Then followed a dry winter, and still drier spring ; the whole amount of rain for the first six months of this year only reaching 6·70 inches, or less than half the usual average for the same period of the year. The sewage in the main drains and its tributaries, both open and covered, were affected by this unusual drought; it stagnated and fermented in its lazy course, and sent its poisonous gases into the dwellings of all, reminding them of their grievous neglect of duty, both towards themselves and their neighbours.

I have said that I was surprised to find such a sudden increase in the number of ague cases at the dispensary, and my surprise was not lessened when, upon investigation, I discovered the cause of the epidemic, which day by day was being developed.

The beginning of this year had been remarkable for its dryness, although the country in this neighbourhood had not suffered from drought ; the crop of hay, although perhaps not so abundant as it would have been had there been more moisture, was considered good, and gathered in without loss. This harvest was succeeded by a beautiful cereal one; what rain fell did so most opportunely, and gladdened the heart of the farmer sufficiently to make him gratefully acknowledge that so splendid a season had not for many years been remembered; nature seemed to smile upon all around her, and would have spread comfort in the homes of the poorest during this joyful time, had not the bright rays of her sun found out the open and secret haunts of filth and improvidence, and rendered the undrained and unventilated cottage unfit to receive the weary labourer and his family after their toil in the harvest fields of their employers. Truly may it be said that the poor cottager loses within his low and confined dormitory the vigorous health which his occupation during the day would ensure him.

I made a house to house visitation in the village and outskirts of Cannington, and subjoin the result of my inquiry.

In the village and immediate neighbourhood, there have been *ninety-four* certified cases amongst a population not amounting to eight hundred souls. Throughout the year, there have been several stray cases occurring from time to time; August, however, was the month in which the epidemic was at its height, as more than forty persons were then suffering from it at one time. In June and July, the greatest portion of other cases took place. The epidemic seems not to have spared one sex more than another, having attacked exactly an equal number of each, viz., forty-seven. The following statistics will give an accurate idea of the time of life most susceptible of the ague-poison.

| | | | | |
|---|---|---|---|---|
| Between 1 | and | 10 years | 21 | cases |
| 10 | „ | 20 | 25 | |
| 20 | „ | 30 | 7 | |
| 30 | „ | 40 | 16 | |
| 40 | „ | 50 | 7 | |
| 50 | „ | 60 | 11 | |
| 60 | „ | 70 | 4 | |
| 70 | „ | 80 | 2 | |
| 80 | „ | 90 | 1 | |
| | | | —94 | |

From these numbers, it will be at once evident that more than three-fourths of the persons affected were capable, on account of their age, of earning wages in an agricultural district.

Above forty persons were laid up in consequence of the ague for more than a month, and many of these had the disease paralysing their strength for two, three, and four months consecutively. The number of cases in each district is as follows:—

No. 1 District. *Nine* cases from the New-road to Oatley; the cottages scattered; some have drains which receive the cottage and the pigsty sewage and have no eject, not even a cesspool. These open receptacles of filth are situated in front of the dwellings, so that the effluvium, which in calm days and nights is described as dreadful, has every opportunity of invading the cottage to which it belongs or the neighbouring one.

No. 2. *Twenty-two* cases in Hornhill, an undulating and elevated locality, on the red sandstone, circumstanced like the latter district with regard to drainage: the cottages, however, are more overcrowded. Pigsties prevalent; in many instances, the pig-soil allowed to accumulate near the water source, and the children in the habit of "going anywhere." Dormitories low, and the windows of the upper rooms near the floor; seldom any fireplaces in the bedrooms.

No. 3. *Fifteen cases.* Cannington-park, Limekiln-lane from Knap to Putnill, elevated and undulating ground, nearly entirely on mountain limestone. The cottages and their drainage exactly similar to the last.

No. 4. *Fifteen cases,* Rodway Hill. On the red sandstone, the north side low and adjoining the marsh; ditches full of stagnant sewage from Rodway farmhouse and adjoining cottages. Accumulated pig's wash and filthy pigsties. The houses on the more elevated portion surrounded by nuisances.

No. 5. *Thirteen cases* in Frog-street, at the bottom of which the drains come to a stand still. The situation is low, on the alluvium, and adjoins the marsh lands.

No. 6 includes the central portion of the village, which gradually rises from the last district, and lies upon the red sandstone and gravel. The number of cases was as follows:—Hennet's-buildings, *one;* Main-street, *seven;* Chit's-lane, *six;* Church-street, *one;* and other places, *five* cases—making in all, with those in other parts, *ninety-four* cases.

The epidemic is happily now on the decline, but relapses are frequent, the same causes still remaining: in consequence of my calling public attention to the above facts, a public meeting was called at the church, the committee of health was requested to resume its duties and carry out my suggestions, viz., to turn the mill stream through the main drain once a week, on Sundays, enlarge the tributary drains, cover those which are now open, and enjoin a periodical simultaneous pumping, so as to wash out the accumulated filth of ages. Up to the present date, not a single step has been taken either publicly or privately to abate the nuisance and remove the cause of disease. The medical man is powerless; he may advise, he may forewarn, but like Cassandra, his prophetic voice is seldom heeded until too late.

